# Meta-analysis of machine learning models for the diagnosis of central precocious puberty based on clinical, hormonal (laboratory) and imaging data

**DOI:** 10.3389/fendo.2024.1353023

**Published:** 2024-03-25

**Authors:** Yilin Chen, Xueqin Huang, Lu Tian

**Affiliations:** ^1^ Department of Thoracic Surgery, Chongqing General Hospital, Chongqing Medical University, Chongqing, China; ^2^ Department of Radiology, Children’s Hospital of Chongqing Medical University, National Clinical Research Center for Child Health and Disorders, Ministry of Education Key Laboratory of Child Development and Disorders, Chongqing Key Laboratory of Pediatric Metabolism and Inflammatory Diseases, Chongqing, China

**Keywords:** machine learning, central precocious puberty, meta-analysis, ML, CPP

## Abstract

**Background:**

Central precocious puberty (CPP) is a common endocrine disorder in children, and its diagnosis primarily relies on the gonadotropin-releasing hormone (GnRH) stimulation test, which is expensive and time-consuming. With the widespread application of artificial intelligence in medicine, some studies have utilized clinical, hormonal (laboratory) and imaging data-based machine learning (ML) models to identify CPP. However, the results of these studies varied widely and were challenging to directly compare, mainly due to diverse ML methods. Therefore, the diagnostic value of clinical, hormonal (laboratory) and imaging data-based ML models for CPP remains elusive. The aim of this study was to investigate the diagnostic value of ML models based on clinical, hormonal (laboratory) and imaging data for CPP through a meta-analysis of existing studies.

**Methods:**

We conducted a comprehensive search for relevant English articles on clinical, hormonal (laboratory) and imaging data-based ML models for diagnosing CPP, covering the period from the database creation date to December 2023. Pooled sensitivity, specificity, positive likelihood ratio (LR+), negative likelihood ratio (LR-), summary receiver operating characteristic (SROC) curve, and area under the curve (AUC) were calculated to assess the diagnostic value of clinical, hormonal (laboratory) and imaging data-based ML models for diagnosing CPP. The I^2^ test was employed to evaluate heterogeneity, and the source of heterogeneity was investigated through meta-regression analysis. Publication bias was assessed using the Deeks funnel plot asymmetry test.

**Results:**

Six studies met the eligibility criteria. The pooled sensitivity and specificity were 0.82 (95% confidence interval (CI) 0.62-0.93) and 0.85 (95% CI 0.80-0.90), respectively. The LR+ was 6.00, and the LR- was 0.21, indicating that clinical, hormonal (laboratory) and imaging data-based ML models exhibited an excellent ability to confirm or exclude CPP. Additionally, the SROC curve showed that the AUC of the clinical, hormonal (laboratory) and imaging data-based ML models in the diagnosis of CPP was 0.90 (95% CI 0.87-0.92), demonstrating good diagnostic value for CPP.

**Conclusion:**

Based on the outcomes of our meta-analysis, clinical and imaging data-based ML models are excellent diagnostic tools with high sensitivity, specificity, and AUC in the diagnosis of CPP. Despite the geographical limitations of the study findings, future research endeavors will strive to address these issues to enhance their applicability and reliability, providing more precise guidance for the differentiation and treatment of CPP.

## 1 Introduction

Central precocious puberty (CPP), also known as true precocious puberty, is characterized by the premature activation of the hypothalamic-pituitary-gonadal (HPG) axis, resulting in clinical pubertal symptoms in girls under 8 years old and boys under 9 years old (1–3). Several countries have conducted research on the prevalence of precocious puberty within their own borders, with findings indicating an increasing trend in childhood precocious puberty. However, consensus regarding the underlying reasons for this rise remains elusive (4–6). Previous studies have indicated a much higher likelihood of girls developing idiopathic central precocious puberty compared to boys. However, a recent nationwide multicenter study from Italy suggests that the percentage of idiopathic forms of CPP in boys may be much higher than previously reported (7). CPP can potentially impact adult height and may even lead to social and psychological disturbances. Notably, girls with CPP face an elevated risk of developing breast or cervical cancer (8, 9). Consequently, timely diagnosis and treatment are crucial for girls with CPP. Meanwhile, peripheral precocious puberty (PPP), clinically known as pseudoprecocious puberty, exhibits clinical features similar to CPP but without activation of the hypothalamic-pituitary-gonadal (HPG) axis (1). In routine clinical diagnosis, diagnosing CPP is challenging without the gonadotropin-releasing hormone (GnRH) stimulation test. However, this test is not only expensive and time-consuming but also often causes anxiety in patients due to the need for establishing vascular access and collecting multiple blood samples at various intervals (10, 11). Furthermore, in non-tertiary or community hospitals with limited resources, this laborious test is not consistently accessible. Therefore, some studies have attempted to explore other convenient clinical, hormonal (laboratory) and imaging-related markers, such as basal sex hormone levels, pelvic ultrasound, or bone age, to identify patients with idiopathic CPP (12–15). However, the cut-off values of these factors varied widely, and their efficiency remains unclear. In recent years, with the advancement of artificial intelligence (AI) in the medical field, clinical, hormonal (laboratory) and imaging data-based machine learning (ML) models have utilized clinical, hormonal (laboratory) and imaging data as inputs to create classifiers, enabling the rapid identification of CPP. This approach offers a new perspective for an objective, swift, and intelligent diagnosis of CPP. However, the results of these studies varied widely and were difficult to directly compare, mainly due to diverse ML methods (16–21). Therefore, the diagnostic value of clinical, hormonal (laboratory) and imaging data-based ML models for CPP remains elusive. The aim of this study was to investigate the diagnostic value of ML models based on clinical, hormonal (laboratory) and imaging data for CPP through a meta-analysis of existing studies.

## 2 Materials and methods

This meta-analysis conformed to the recommendations provided in the Cochrane Handbook for Systematic Reviews of Diagnostic Test Accuracy (22) and adhered to the guidelines set by the Preferred Reporting Items for Systematic Reviews and Meta-Analyses (PRISMA) (23).

### 2.1 Search strategy

A comprehensive search was performed using PubMed, EMBASE, The Cochrane Library, Wiley Online Library, and the Web of Science to identify relevant articles in English (database creation dated to December 2023).The search strategy adhered to the Population, Intervention, Comparison, Outcome, and Study Design (PICOS) principle (24) (P: “CPP”, I: “ML, DL”, S: “diagnostic test”). The search employed a blend of Medical Subject Heading (MeSH) terms and free-text terms, as follows:(“central precocious puberty” [MeSH] or “CPP” [text]) and (“machine learning” [MeSH] or “ML” [text] or “deep learning” [text] or “DL” [text]) and (“sensitivity and specificity” [MeSH] or predict* [text] or diagnos* [text] or accura* [text]).

### 2.2 Selection criteria

Included studies were those that employed machine learning models utilizing clinical, hormonal (laboratory) and imaging data for the diagnosis of CPP, with the capability to formulate a 2×2 table encompassing true positive (TP), false positive (FP), true negative (TN), and false negative (FN) values. Studies lacking a clearly defined reference standard or providing insufficient data for the computation of study outcomes were excluded. Animal experiments, case reports, meta-analyses, and reviews were also excluded.

### 2.3 Study selection and data extraction

The initial screening involved evaluating study titles and abstracts based on the inclusion criteria before proceeding to a comprehensive review of the full text. Two researchers independently conducted data extraction, and any disagreements were resolved through mutual consultation or discussion with a third expert. Extracted data encompassed details such as authorship, publication year, gender, CPP and non-CPP group size and age, features utilized, classifier employed, optimal classifier, and results (specificity, sensitivity, and AUC). In cases where additional information was required, corresponding authors were contacted.

### 2.4 Quality of the studies

The evaluation of the risk of bias in individual studies was conducted employing the Quality Assessment of Diagnostic Accuracy Studies-2 (QUADAS-2) checklist (25). Independent reviewers assessed each article according to these criteria, and any disparities were resolved through discussion.

### 2.5 Statistical analysis

Stata software (version 23; Stata Corporation, College Station, Texas, USA) was utilized for both graphical representation and calculations. Pooled sensitivity (SEN), pooled specificity (SPE), positive likelihood ratio (LR+), negative likelihood ratio (LR-), diagnostic odds ratio (DOR) with a 95% confidence interval (CI), summary receiver operating characteristic (SROC) curve, and area under the curve (AUC) were computed to evaluate the accuracy of clinical, hormonal (laboratory) and imaging data-based ML models in diagnosing CPP. A binary generalized linear mixture model was employed for pooling. Heterogeneity was assessed using chi-square and Cochran Q tests, with I^2^ > 50% indicating substantial heterogeneity. Subgroup analyses and meta-regression analyses were carried out to investigate potential sources of heterogeneity. Publication bias was assessed using Deeks’ funnel plot asymmetry test, with significance set at P < 0.05.

## 3 Results

### 3.1 Literature search results

**Figure 1:**
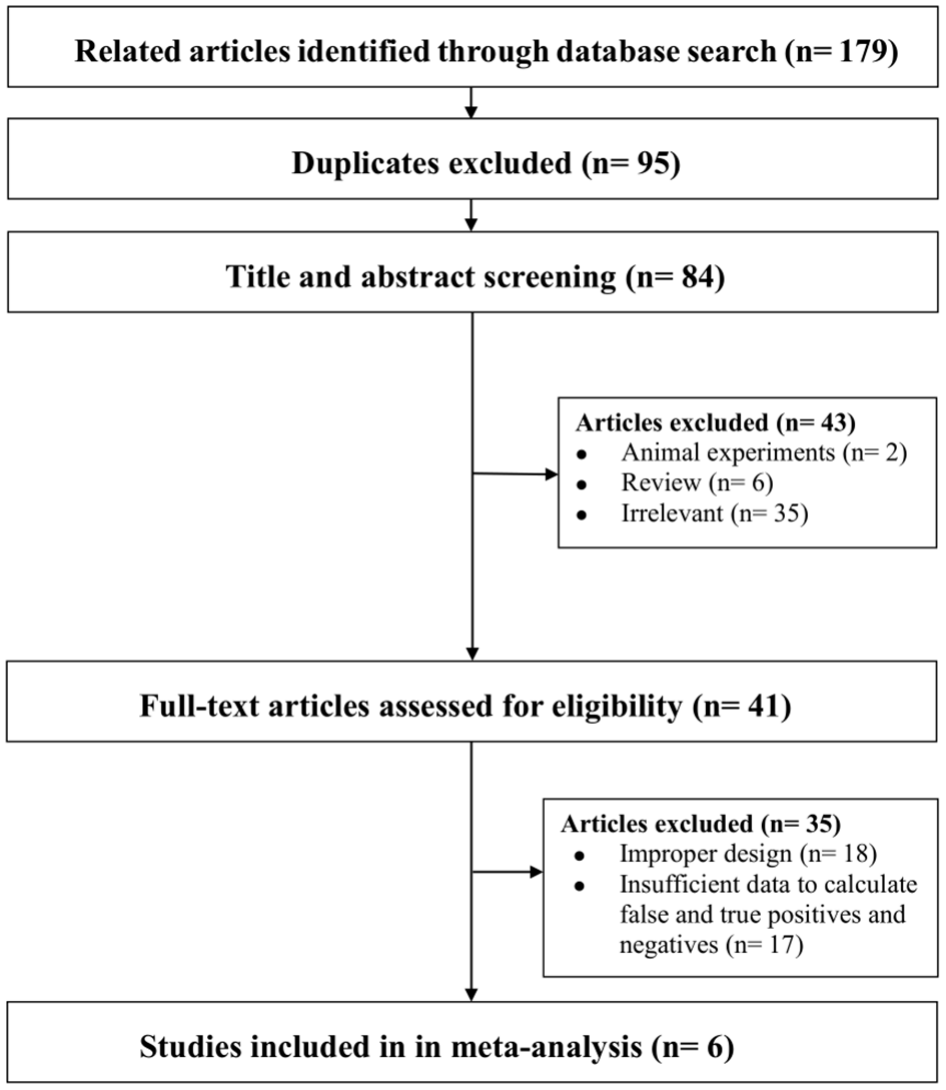
Flowchart summarizing the study selection process. Note-Some studies were excluded for more than one reason. Irrelevant: Did not investigate the diagnostic value of ML models based on clinical, hormonal (laboratory) and imaging data for CPP. Improper design: Did not meet the selection criteria.

Through database searches, a total of 179 articles were initially identified. Subsequently, 95 duplicate articles were excluded, followed by the exclusion of 43 articles after reviewing titles and abstracts. After a thorough full-text review, 35 additional studies were excluded, resulting in the inclusion of 6 studies for our analysis (refer to **Figure 1**).

### 3.2 Characteristics of the included studies

**Table 1** presents the characteristics of the included studies. Notably, all analyzed articles were recently published, with the earliest study dating back to 2019, highlighting the relatively recent introduction of machine learning models in the context of CPP diagnosis. Examining **Table 1** reveals a singular focus on girls across all the studies. The CPP group exhibited case numbers ranging from 137 to 1153, while the non-CPP group ranged from 24 to 1370 cases. It is noteworthy that among the included studies, only Zou et al. (21) reported subjects with PPP as controls. In the other studies, the non-PPP group was represented by subjects who tested normal on diagnostic tests. In Pan et al. (19), reference was made to a self-administered questionnaire administered in schools. The average age of most cases hovered around 7 years old. Furthermore, there was substantial variation in ML methods among the studies, particularly in terms of feature selection and classifier choice. The data sources, extracted from clinical, hormonal (laboratory) and imaging data, encompassed four main aspects: general features (e.g., age, height, weight, body mass index), clinical features (e.g., disease duration, breast Tanner stage, vaginal bleeding), laboratory features (e.g., 17α-hydroxyprogesterone (17α-hydroxy), adrenocorticotropic hormone (ACTH), cortisol, human chorionic gonadotropin (HCG), follicle-stimulating hormone (FSH), luteinizing hormone (LH), prolactin, estradiol (E2), total testosterone (TT)), and imaging features (e.g., bone age X-rays, pelvic ultrasound, pituitary MRI imaging features). Additionally, the classifiers used in the studies were diverse, including logistic regression (LR), support vector machine (SVM), Gaussian naive Bayes (GaussianNB), extreme gradient boosting (XGBoost), random forest (RF), and k-nearest neighbor algorithm (kNN), among others. Finally, the levels of AUC (ranging from 0.79 to 0.97), sensitivity (ranging from 0.34 to 0.96), and specificity (ranging from 0.77 to 0.93) varied across ML models, indicating the need for further pooled analysis to comprehensively assess the diagnostic value of clinical, hormonal (laboratory) and imaging data-based ML models for CPP.

**Table 1:** Characteristics of the included studies.

Author	Year	Sex (F/M)	CPP group		Non-CPP group		Feature	Classifier	Optimal classifier	SPE	SEN	AUC
			N	Age (mean ± SD), y	N	Age (mean ± SD), y						
Pan et al. (16)	2020	F	1153	7.056 ± 1.13	1370	7.476 ± 1.09	General features, Clinical features, Laboratory features, BA,US	XGBoost	XGBoost	77.88	85.71	0.88
Huynh et al. (17)	2022	F	524	7.2 ± 1.8	90	7.5 ± 1.5	General features, Clinical features, Laboratory features, BA	kNN, GNB, LR, RF, XGBoost	RF	89.30	96.60	0.97
Pang et al. (18)	2022	F	408	10.9	5119	10.8	General features, Clinical features, Laboratory features	LR,DT, Adaboost, SVM, RF,kNN,GBM,GNB,et al	GBM	93.22	34.39	0.79
Pan et al. (19)	2019	F	791	7.52 ± 0.99	966	7.07± 1.11	General features, Clinical features, Laboratory features	XGBoost,RF,SVM,DT	XGBoost	85.39	77.94	0.89
Chen et al. (20)	2023	F	137	8.51	24	8.55	General features, Clinical features, Laboratory features	LR, RF, GBM, XGBoost	LR	85.7	95.2	0.88
Zou et al. (21)	2023	F	185	7.52± 0.56	307	7.21± 0.76	General features, Clinical features, Laboratory features US, MRI Radiomics	RF, DT, SVM, GNB, LR	LR	85.7	72.7	0.86

F, female; M, male; N, number of patients; CPP, central precocious puberty; BA, bone age; US, ultrasonography; MRI, magnetic resonance imaging; XGBoost, extreme gradient boosting; kNN, k- nearest neighbor algorithm; GNB, gaussian naive bayes; LR, logistic regression; RF, random forest; DT, decision tree; SVM, support vector machine; GBM, gradient boosting machine; SPE, Specificity; SEN, Sensitivity; AUC, area under the curve.

### 3.3 Quality of the studies

**Table 2** provides an overview of the risk of bias and applicability concerns identified in the included studies. The information is categorized into 14 items, distributed across four sections: patient selection, index test, reference standard, and flow and timing. While none of the studies met all the items, each study fulfilled a minimum of 10 items. Notably, high-risk items were mainly reflected in the patient selection part because the included studies were case-control studies rather than randomized controlled trials, and it is not clear whether the sample of patients enrolled is a continuous case. whereas the remaining sections indicated a low risk of bias.

**Table 2:** Risk of bias and applicability concerns summary of the included studies.

Study	Risk of bias^1^				Applicability^2^		
	Patient selection^3^	Index text^4^	Reference Standard^5^	Flow and timing^6^	Patient selection	Index text	Reference standard
Pan et al (16)	H	L	L	L	L	L	L
Huynh et al. (17)	H	L	L	L	L	L	L
Pang et al. (18)	H	L	L	L	L	L	L
Pan et al. (19)	H	L	L	L	L	L	L
Chen et al. (20)	H	L	L	L	L	L	L
Zou et al. (21)	H	L	L	L	L	L	L

H, High risk; L, Low risk; U, Unclear.

^1^Risk of Bias is judged as “low,” “high,” or “unclear.” If the answers to all items questions for a part are “yes,” then risk of bias can be judged low. If any item question is answered “no,” potential for bias exists. The “unclear” category should be used only when insufficient data are reported to permit a judgment.

^2^Applicability sections are structured in a way similar to that of the bias sections but do not include signaling questions. Review authors record the information on which the judgment of applicability is made and then rate their concern that the study does not match the review question.Concerns about applicability are rated as “low,” “high,” or “unclear.” the “unclear” category should be used only when insufficient data are reported.

^3^Part 1: Patient Selection.

Risk of Bias:

item 1: Was a consecutive or random sample of patients enrolled?

item 2: Was a case–control design avoided?

item 3: Did the study avoid inappropriate exclusions?

Applicability:

item 4: Are there concerns that the included patients and setting do not match the review question?

^4^Part 2: Index Test.

Risk of Bias:

item 5: Were the index test results interpreted without knowledge of the results of the reference standard?

item 6: If a threshold was used, was it prespecified?

Applicability:

item 7: Are there concerns that the index test, its conduct, or its interpretation differ from the review question?

^5^Part 3: Reference Standard.

Risk of Bias:

item 8: Is the reference standard likely to correctly classify the target condition?

item 9: Were the reference standard results interpreted without knowledge of the results of the index test?

Applicability:

item 10: Are there concerns that the target condition as defined by the reference standard does not match the question?

^6^Part 4: Flow and Timing.

Risk of Bias:

item 11: Was there an appropriate interval between the index test and reference standard?

item 12: Did all patients receive a reference standard.

item 13: Did all patients receive the same reference standard?

item 14: Were all patients included in the analysis?

### 3.4 Main results

**Figure 2:**
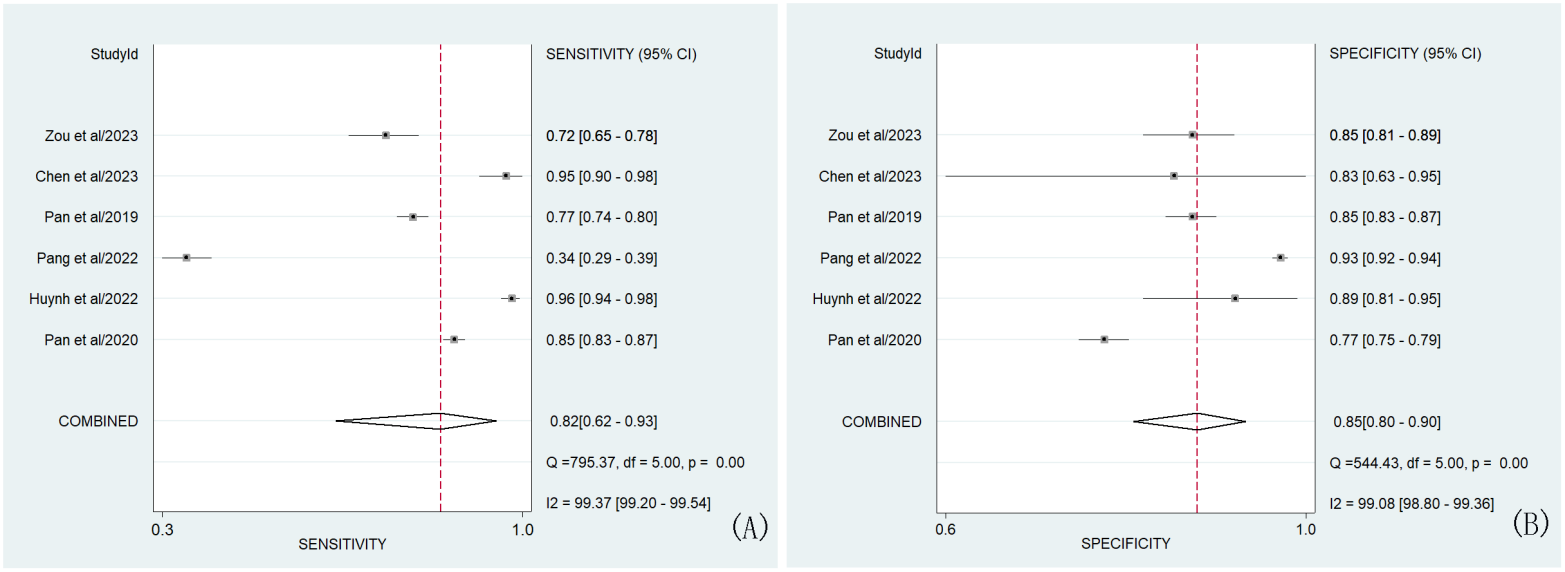
Forest plots of the sensitivity **(A)** and specificity **(B)** of ML models based on clinical, hormonal (laboratory) and imaging data for the diagnosis of CPP. Note-The dots correspond to the individual studies included in this analysis, and both sides of the line represent the 95% confidence interval. The narrower the line is, the greater the accuracy of the study and the greater the weight. The diamond corresponds to the pooled result. The intermediate vertical line represents an invalid line. Q statistic test card square value (chi-square), degree of freedom (df), p-values and I^2^ statistic test results (Inconsistency (I- square)) correspond to heterogeneity test results. The Q test was used to assess heterogeneity, while the I^2^ test was used to measure the size of heterogeneity. Heterogeneity was considered when *p* was less than 0.01. If I^2^<25%, no heterogeneity was noted. If the value of I^2^ was between 25% and 50%, the degree of heterogeneity was considered to be small. If the value of I^2^ was between 50% and 75%, heterogeneity was noted. If I^2^>75%, large heterogeneity was noted.

**Figure 3:**
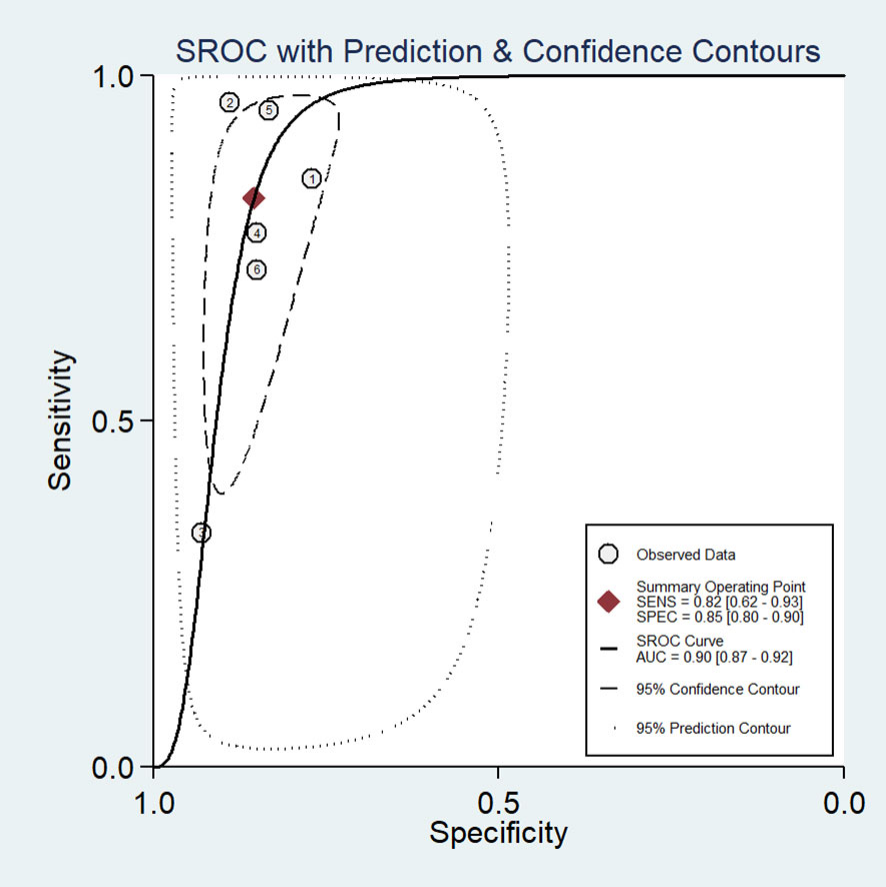
Hierarchical summary SROC plots of ML models based on clinical, hormonal (laboratory) and imaging data for the diagnosis of CPP. The ellipse represents 95% CI for this estimate. Numbers correspond to enrolled studies.

**Figure 4:**
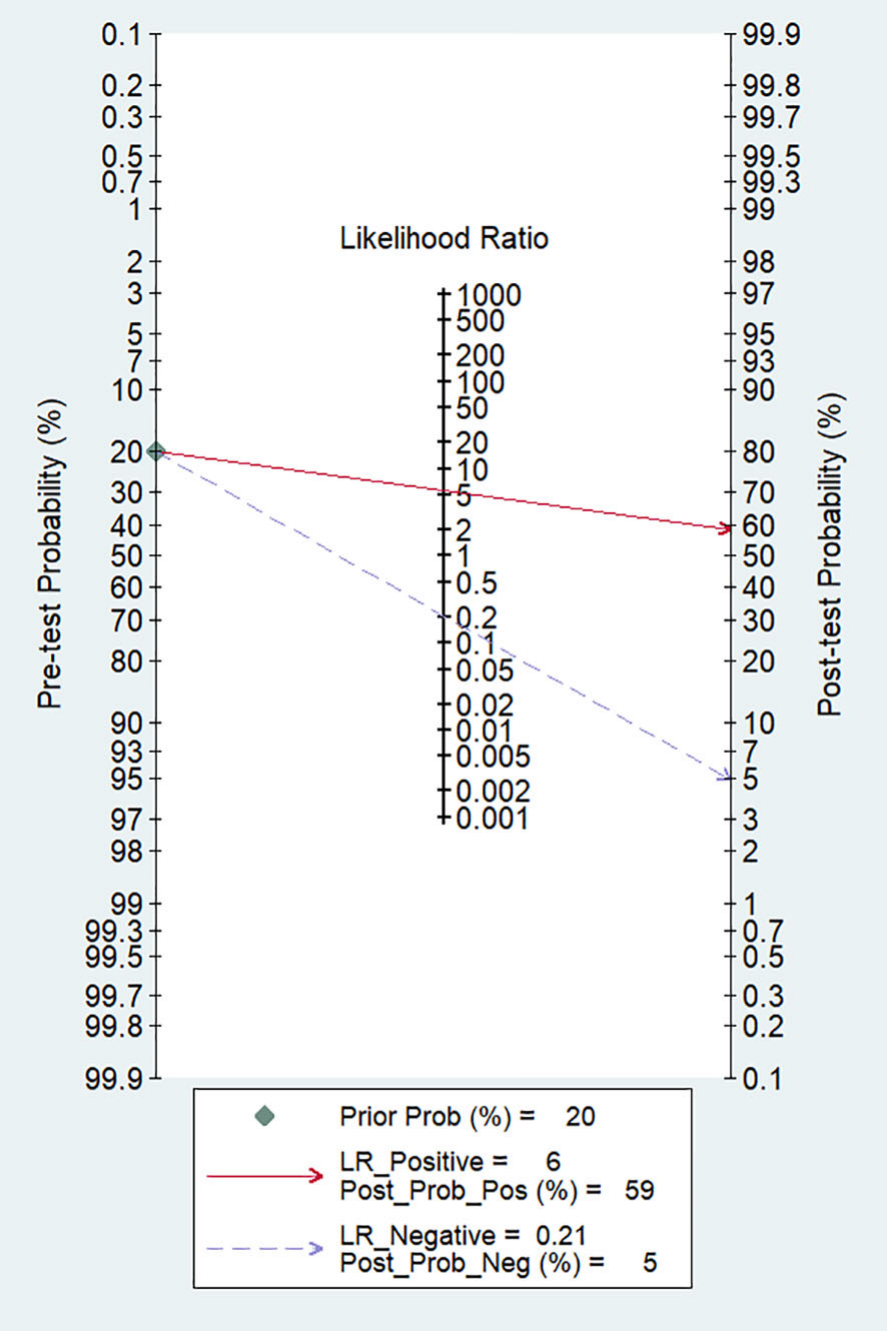
Fagan nomogram of ML models based on clinical, hormonal (laboratory) and imaging data for the diagnosis of CPP.

In our study, the pooled sensitivity and specificity of clinical, hormonal (laboratory) and imaging data-based ML models for diagnosing for CPP were 0.82 (95% CI 0.62-0.93) and 0.85 (95% CI 0.80–0.90), respectively (**Figure 2**). SROC curves showed that the accuracy of the AUC was 0.90 (**Figure 3**). The closer the AUC is to 1, the better the diagnosis test. Based on these findings, clinical, hormonal (laboratory) and imaging data-based ML models has good diagnostic value for CPP and exhibits high sensitivity and specificity. Theoretically, the higher the positive likelihood ratio, the better the diagnostic test is in correctly identifying the true disease. The lower the negative likelihood ratio, the better the ability of the diagnostic test to exclude a disease. As noted in **Figure 4**, clinical, hormonal (laboratory) and imaging data-based ML models had a high positive likelihood ratio (6) and a low negative likelihood ratio (0.21), revealing that clinical, hormonal (laboratory) and imaging data-based ML models exhibited an excellent ability to confirm or exclude CPP.

### 3.5 Publication bias

**Figure 5:**
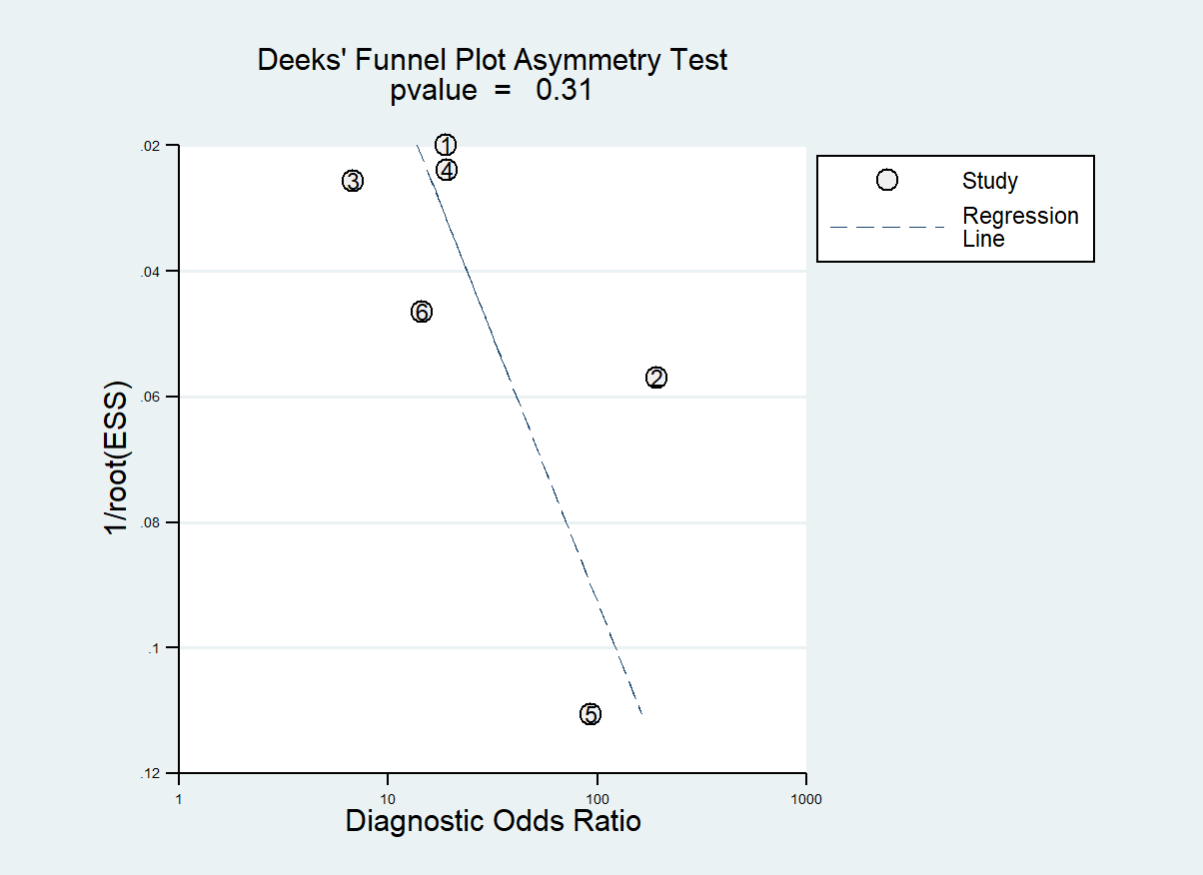
Deeks’ funnel plot asymmetry test for publication bias Note-Numbers correspond to enrolled studies.

We assessed publication bias using Deeks’ regression test of asymmetry (t = 1.17; P = 0.31) (refer to **Figure 5**). Examination of Deeks’ funnel plots for clinical, hormonal (laboratory) and imaging data-based ML models indicated the absence of publication bias (P > 0.05).

### 3.6 Heterogeneity and meta-regression analyses

Significant heterogeneity was observed among the studies (I² = 99.37%, 95% CI 99.20-99.54). To identify the source of heterogeneity, subgroup analyses and meta-regression analysis were conducted (refer to **Table 3**). The primary contributor to heterogeneity appeared to be the variations in features and classifiers. Meta-regression analysis further emphasized that differences in features and classifiers were the key sources of heterogeneity across these studies. Results from the subgroup analysis revealed that: studies incorporating image features exhibited higher sensitivity and specificity compared to those that did not include image features (P < 0.05). Studies employing the LR classifier demonstrated higher sensitivity and specificity in diagnosing CPP compared to those using the XGBoost classifier (P < 0.05). However, studies utilizing the RF classifier displayed higher sensitivity and specificity in diagnosing CPP compared to those opting for the LR model (P < 0.05).

**Table 3:** Univariate and multivariate meta-regression analyses for identifying covariates to explain heterogeneity among studies on clinical, hormonal (laboratory) and imaging data-based ML models for the diagnosis of CPP.

Covariates	Multivariate meta-regression			SEN and 95% CI	SPE and 95% CI
	LR (Chi-square test)	*P*	I^2^ index (%)		
**Feature** Image feature (n =4) Non-image feature (n = 2)	6.13	0.04	67	0.90 [0.83 - 0.97] 0.53 [0.25 - 0.81]	0.90 [0.86 - 0.94] 0.83 [0.77 - 0.89]
**Classifier** LR (n =4) XGBoost (n = 4)	4.22	0.02	53	0.93 [0.87 - 0.99] 0.62 [0.42 - 0.82]	0.88 [0.84 - 0.93] 0.82 [0.73 - 0.90]
**Classifier** RF (n =5) LR (n = 4)	4.68	0.01	57	0.91 [0.87 - 0.95] 0.77 [0.47 - 1.00]	0.91 [0.87 - 0.95] 0.81 [0.77 - 0.86]
**Classifier** GBM (n =1) RF (n = 5)	2.99	0.22	33	–	–

LR, logistic regression; XGBoost, extreme gradient boosting; RF, random forest; GBM, gradient boosting machine; SPE, Specificity; SEN, Sensitivity. P value of <0.05 was considered statistically significant.

## 4 Discussion

To the best of our knowledge, this is the first meta-analysis in existing research on ML models for diagnosing CPP based on clinical, hormonal (laboratory) and imaging data. Our meta-analysis reveals that ML models utilizing clinical, hormonal (laboratory) and imaging data for CPP diagnosis demonstrate high AUC, sensitivity, and specificity, indicating significant diagnostic value. This suggests that the application of ML models in diagnosing CPP holds promising potential. The development of this technology is attributed to the advancements in AI in the medical field. ML models based on clinical, hormonal (laboratory) and imaging data utilize clinical, hormonal (laboratory) and imaging data as inputs for ML algorithms, creating classifiers that rapidly identify CPP. This provides a new perspective for an objective, swift, and intelligent diagnosis of CPP.

In the studies we included, despite the use of different ML models for diagnosing CPP, the steps involved in ML technology were similar, primarily encompassing three stages: feature selection, feature extraction, and decision-making through a classifier. On the one hand, feature selection is a critical component of ML technology. The selection of features primarily derives from clinical, hormonal (laboratory) and imaging data, such as age, gender, height, weight, breast development, vaginal bleeding, LH, FSH, E2, TT, bone age, pelvic ultrasound, pituitary MRI, etc. In the studies we included, while the types and number of features varied, all studies incorporated LH and FSH as laboratory indicators, indicating their significant discriminatory value for CPP, consistent with previous research (26–28). Certainly, we aim to include as few features as possible to establish a machine learning model that can accurately diagnose CPP. Pan et al. (19) employed ML algorithms, incorporating 19 features such as age, baseline LH, baseline FSH, insulin-like growth factor-1 (IGF-1), growth hormone (GH), etc., to construct a predictive model for CPP diagnosis. They achieved an AUC range of 0.88 to 0.90, with sensitivity ranging from 77.91% to 77.94% and specificity from 84.32% to 87.66%. Huynh et al. (17) also developed six classical ML diagnostic models for girls suspected of having CPP, including 14 clinical indicators such as baseline LH, baseline FSH, uterine volume, etc. The best-performing model, the RF model, demonstrated an AUC of 0.972, sensitivity of 96.6%, and specificity of 89.3%. It can be observed that compared to the study by Pan et al., Huynh et al. achieved improved diagnostic efficiency while reducing the number of included features. Our meta-analysis does not provide definitive recommendations for the development of an optimal feature combination. In high-dimensional spaces, there is typically no method superior to others. However, our subgroup analysis results indicate that studies incorporating imaging features tended to have higher sensitivity and specificity in diagnosing CPP compared to studies that did not include imaging features. Imaging features primarily include BA, pelvic ultrasound, pituitary MRI, etc. Pan et al. (19) demonstrated that using only laboratory data is challenging to effectively differentiate between CPP and non-CPP (sensitivity of 66.23%). However, once combined with pelvic ultrasound, the model showed a significant improvement in the area under the ROC curve, increasing from 0.42 to 0.63. Therefore, it is suggested that in the development of machine learning diagnostic models for CPP, efforts should be made to include imaging features whenever possible. On the other hand, classifier selection is another crucial aspect of machine learning technology. The included studies encompassed various types of classifiers, including XGBoost, RF, GBM, LR, SVM, naïve Bayesian, kNN, etc. In the studies we incorporated, apart from Chen et al. (20) and Zou et al. (21), where LR was the optimal model, the optimal models in other studies varied, including XGBoost, RF, GBM, etc. Our subgroup analysis results indicate that studies opting for the RF classifier tended to have higher specificity and sensitivity compared to studies choosing other types of classifiers. Therefore, it is recommended that in the development of machine learning diagnostic models for CPP, efforts should be made to include the RF classifier whenever possible.

We hope that the results of the aforementioned meta-analysis will catalyze further advancements in this field. Despite the challenging nature of diagnosing CPP using ML models based on clinical, hormonal (laboratory) and imaging data, and being in its early stages, our meta-analysis suggests that these technologies have shown promising results. Future research should focus on developing ML models that incorporate fewer features, provide accurate diagnoses, and are interpretable. We further observed that Zou et al. (21) employed the SHAP global interpretability technique, and Huynh et al. (17) utilized the LIME analysis method to interpret their machine learning models. This interpretability is crucial for clinical decision-making as it enhances healthcare professionals’ trust in the model outputs and helps them better understand the reasons behind specific diagnostic decisions. This breaks the curse of machine learning models being perceived as black boxes. Additionally, in clinical practice, these machine learning models can be developed into a small software tool to facilitate their application in healthcare settings. In resource-limited medical environments, primary care physicians can use the model’s results to make preliminary management decisions. If, based on the model’s assessment, a patient is classified into the CPP group, it suggests a reason to suspect early development, warranting appropriate medical intervention.

We assessed the quality of the included studies using the following four components: patient selection, index test, reference standard, and flow and timing, among which the high risk was mainly reflected in the patient selection component. A potential explanation for this finding is that the inclusion criteria for diagnostic trials are often based on case-control trials rather than randomized controlled trials, and patients included in the study only reported the time period without specifying whether they were consecutive cases. In the included studies, ML methods and the gold standard method were performed without knowing the results of each other, and the GnRH stimulation test was used as the gold standard. Therefore, selection bias was minimal, and the results were reliable, indicating that these factors were associated with a low risk of bias. Additionally, the Deeks funnel plot showed no publication bias in these studies.

In the included studies, a significant amount of heterogeneity was observed, and the causes of this heterogeneity were multifaceted. Differences in feature selection, imaging methods, and classifiers were the main contributors to this heterogeneity. However, in another sense, this heterogeneity might be valuable when developing clinically deployable ML models. To accurately reflect the actual performance of ML models in diagnosing CPP, these models must be tested on different features, imaging methods, and classifiers to identify the model with the best performance. In addition, differences in control groups also serve as a primary source of heterogeneity. Among the included studies, only Zou et al. (21) reported subjects with PPP as controls. In the other studies, the non-PPP group was represented by subjects who tested normal on diagnostic tests. In Pan et al. (19), reference was made to a self-administered questionnaire administered in schools. These factors may all contribute to the heterogeneity.

Our study has some limitations. Our study has several limitations. Firstly, all participants were recruited from China and Taiwan, which may restrict the generalizability of our findings as environmental factors, ethnicity, and medical conditions can vary significantly across different regions. Therefore, caution should be exercised when extrapolating our results to other populations, and future research should consider geographical, ethnic, and medical variations to enhance the applicability of our findings. Secondly, distinguishing between rapidly progressing and slowly progressing forms of central precocious puberty (CPP) poses a significant challenge for pediatricians, and our meta-analysis failed to provide clear guidance in this regard. Thus, future research should prioritize addressing this issue by exploring methods to differentiate between different presentations of CPP and guiding personalized treatment strategies. Lastly, the substantial heterogeneity observed among the included studies may impact the evaluation of the diagnostic performance of machine learning (ML) models. Given the limited number of studies and the ongoing development of ML technology, conducting further research in this field is crucial for accurately assessing the utility of ML in diagnosing CPP.

## 5 Conclusions

In summary, our meta-analysis findings demonstrate promisingly high accuracy of machine learning models based on clinical, hormonal (laboratory), and imaging data in diagnosing CPP, exhibiting elevated levels of sensitivity, specificity, and AUC values. Nevertheless, we acknowledge certain limitations regarding the generalizability of our results due to the exclusive inclusion of participants from China and Taiwan. Given the disparities in environmental factors, ethnicity, and healthcare conditions across different regions, caution should be exercised when extrapolating these findings to other populations. Additionally, while distinguishing between rapid and slow progression forms of CPP remains a challenge for pediatricians, we are optimistic about future research prospects. Future studies will continue to address these challenges to further enhance the applicability and reliability of the results, and explore more effective means of differentiating between various types of CPP, thereby providing more precise guidance for appropriate treatment strategies.

## Data Availability

The original contributions presented in the study are included in the article/**Supplementary Material**. Further inquiries can be directed to the corresponding author.
