# Corrigendum: Meta-analysis of machine learning models for the diagnosis of central precocious puberty based on clinical, hormonal (laboratory) and imaging data

**DOI:** 10.3389/fendo.2025.1579425

**Published:** 2025-03-05

**Authors:** Yilin Chen, Xueqin Huang, Lu Tian

**Affiliations:** ^1^ Department of Thoracic Surgery, Chongqing General Hospital, Chongqing Medical University, Chongqing, China; ^2^ Department of Radiology, Children’s Hospital of Chongqing Medical University, National Clinical Research Center for Child Health and Disorders, Ministry of Education Key Laboratory of Child Development and Disorders, Chongqing Key Laboratory of Pediatric Metabolism and Inflammatory Diseases, Chongqing, China

**Keywords:** machine learning, central precocious puberty, meta-analysis, ML, CPP

In the published article, there was an error in affiliation(s) 1. Instead of “Chongqing University”, it should be “Chongqing Medical University”.

The authors apologize for this error and state that this does not change the scientific conclusions of the article in any way. The original article has been updated.

